# Plasma pooling in combination with amotosalen/UVA pathogen inactivation to increase standardisation and safety of therapeutic plasma units

**DOI:** 10.1111/tme.12763

**Published:** 2021-03-08

**Authors:** Michal Bubinski, Agnieszka Gronowska, Pawel Szykula, Kamila Kluska, Ilona Kuleta, Emilia Ciesielska, Marcus Picard‐Maureau, Elzbieta Lachert

**Affiliations:** ^1^ Regional Blood Transfusion Center Lodz Poland; ^2^ Medical Laboratory SYNEVO Lodz Poland; ^3^ Cerus Europe B.V. Amersfoort The Netherlands; ^4^ Institute of Hematology and Transfusion Medicine Warsaw Poland

**Keywords:** amotosalen/UVA, pathogen inactivation, plasma pooling, plasma standardisation

## Abstract

**Objectives:**

Assessment of the impact of pooling five single‐donor plasma (SDP) units to obtain six pathogen‐reduced therapeutic plasma (PTP) units on standardisation and the retention of labile coagulation factors.

**Background:**

SDP shows a high inter‐donor variability with potential implications for the clinical treatment outcome. Additionally, there is still an existing risk for window‐period transmissions of blood borne pathogens including newly emerging pathogens.

**Methods/Materials:**

Five ABO‐identical SDP units were pooled, treated with the INTERTCEPT™ Blood System (Cerus Corporation, U.S.A.) and split into six PTP units which were frozen and thawed after 30 days. The variability in volume, labile coagulation factor retention and activity was assessed.

**Results:**

The variability of volumes between the PTP units was reduced by 46% compared to SDP units. The variability in coagulation factor content between the PTP units was reduced by 63% compared to SDP units. Moderate, but significant losses of coagulation factors (except for vWF) were observed in PTPs compared to SDPs.

**Conclusion:**

The pooling of five SDP units to obtain six PTP units significantly increases product standardisation with potential implications for safety, economics as well as transfusion‐transmitted pathogen safety, making it an interesting alternative to quarantine SDP (qSDP) and pathogen‐reduced SDP.

## 1 INTRODUCTION

The total protein profile (including contents of coagulation factors) in individual fresh frozen plasma (FFP) units shows a high variability due to the impact of genetic factors on the human proteome.^1^ Additionally, individual levels of coagulation factors depend on the plasma collection methods and procedures^2^ as well as on the blood group. In Poland, most therapeutic plasma units are produced from individual whole‐blood donations (89% in 2018). Despite the implementation of automation for whole blood separation there are significant differences in volume between plasma units, which also depend on the individual manufacturing methods. High individual variations in coagulation factor content in plasma may affect treatment outcome and render the therapeutic effect of transfusion less predictable. Plasma pooling to overcome individual coagulation factor variations and volume variations may be a potential solution. However, plasma pooling without any pathogen inactivation procedure also bears an increased risk of pathogen transmission. For that reason, plasma pooling alone was not recommended in Poland. Despite the gradual implementation of safety measures like restrictive donor selection, improved blood collection procedures and the introduction of highly sensitive tests, the highest number of HCV window period infections of blood donors among European countries was noted in Poland since the implementation of HCV NAT screening through 2008.^3^ The frequency of HIV infection among Polish blood donors is relatively high as compared to other developed countries. A significant part of HIV positive donors (22.8%) where infected less than 100 days before donation (Fiebig stage I–V). Currently, the residual risk of donations in the diagnostic window period in Poland is higher for HIV than for HBV and HCV (Piotr Grabarczyk, unpublished data). To address concerns regarding the transmission of blood borne viruses, the requirement to conduct either quarantine of SDP until second negative test of the donor (qSDP) or pathogen inactivation‐treatment (PI‐SDP) was introduced in Poland already in 2008.^4^ Currently the riboflavin/UVB light pathogen reduction system (Mirasol™), the methylene blue/visible light (Theraflex™ MB) and amotosalen/UVA light (INTERCEPT™ Blood System) pathogen inactivation systems are used in Polish blood centres. 10.23% of plasma units in Poland were pathogen‐reduced in 2018.^4^ Additionally, SD‐plasma (solvent‐detergent, Octaplas™) is available through pharmacies. In the light of newly emerging pathogens (Dengue Virus, West‐Nile Virus, Zika Virus) and the current COVID‐19 pandemic, the concept of qSDP is becoming questionable. The majority of infected individuals shows no symptoms, despite positive NAT results.^5^, ^6^, ^7^, ^8^ SARS‐CoV‐2 (the virus causing COVID‐19) genomic RNA has been detected in blood products from asymptomatic donors.^9^ Evidence for transmission by blood transfusion has not been shown yet but cannot be excluded.^10^ The current situation shows that the qSDP concept only works for specific pathogens if testing is applied, which is not the case for SARS‐CoV‐2 as well as potentially newly emerging pathogens in the future.

Amotosalen/UVA (AS) pathogen inactivation technology (INTERCEPT™ Blood System, Cerus Corporation), a targeted photochemical reaction irreversibly crosslinking nucleic acids,^11^ allows the treatment of pools of five plasma units and splitting into six standardised therapeutic units.^12^ The key objectives for this production method are the prevention of transmission of infectious agents and the preservation of the haemostatic capacity as well as the clinical effectiveness. AS‐treatment of plasma effectively inactivates a broad spectrum of pathogens, including newly emerging pathogens.^13^, ^14^ In vitro studies revealed a moderate loss of coagulation factors post PI‐treatment, with an overall retention meeting the regulatory criteria for therapeutic use.^15^, ^16^ Also, plasma treated with the INTERCEPT Blood System has been analysed in a series of clinical studies including patients with acquired and inherited coagulopathies as well as patients requiring therapeutic plasma exchange. In all these studies, the plasma showed a high level of tolerability and a safety profile comparable to conventional plasma.^17^, ^18^

Our Institute, the Lodz Regional Blood Transfusion Center, is a Polish mid‐size blood centre with almost 61.000 whole blood donations in 2018. We produced almost 61.000 plasma units (30% of them for clinical use, 6% of those pathogen‐reduced) in 2018. In the current study, we intended to evaluate the impact of pooling five SDP units to obtain six PTP units and AS pathogen inactivation treatment on plasma standardisation and the content of labile coagulation factors as a potential alternative to qSDP and PI‐SDP to potentially improve the clinical safety of our plasma products.

## 2 MATERIAL AND METHODS

### 2.1 *Whole blood collection and plasma preparation*

Whole blood units (450 ± 50 ml) were collected from voluntary donors at the Regional Blood Transfusion Center (RBTC) in Lodz using Compoflow™ containers (Fresenius Kabi, Germany) with citrate/phosphate/dextrose solution (CPD) as anticoagulant. The whole blood units were stored for 2 h at room temperature (RT) followed by centrifugation at 2699×*g* for 11 min at 22° and subsequent separation into red cells, buffy coat and plasma with a CompoMat™ G5 Automated Blood Component Separator (Fresenius Kabi). All units of whole blood were tested negatively for anti‐HIV1/2, anti‐HCV, HBsAg, HIV‐RNA, HCV‐RNA, HBV‐DNA and *Treponoma Pallidum* antibodies according to Polish guidelines.

### 2.2 *Plasma pooling and pathogen inactivation*

Five fresh ABO‐identical SDP units were pooled with an Optipool DONOpack™ Plasma Pooling Set (Cerus Corporation) according to the manufacturer's instructions at RT <4 h post collection. After mixing, the pool was divided into two equal weight minipools. Each minipool was treated with the INTERCEPT™ Processing Set for Plasma (Cerus Corporation) using the INTERCEPT™ Illuminator INT 100 (Cerus Corporation) according to the manufacturer's instructions. Residual amotosalen and photoproducts were removed with the built‐in compound adsorption device. Each minipool was subsequently divided into three storage bags resulting in six PTP units. The plasma units were frozen using a shock freezer (MABAG, Germany) within 8 h post collection as FFP and stored at ≤−25°C according to Polish guidelines.

### 2.3 *Sampling and analytical testing*

Samples were collected from the SDPs before pooling, from the pools directly after pooling and mixing (pre‐inactivation) and from the PTP units immediately after pathogen inactivation, into Eppendorf®Safe‐Lock microcentrifuge tubes (volume 1.5 ml). Samples were frozen at −30°C and stored for 30 days. All samples were thawed simultaneously at 37°C in a water bath and analysed at the same time for each assay with an ACL TOP 500 Haemostasis Testing System (Werfen, Spain). Prothrombin Time (PT) was analysed with a HemosIL RecombiPlasTin 2G kit (Werfen). Activated Partial Thromboplastin Time (APTT) was analysed with an APTT‐SP (liquid) kit (Werfen). Fibrinogen activity was analysed with a HemosIL Q.F.A Thrombin (Bovine) kit (Werfen), based on the Clauss method. Factor VIII (FVIII) activity was analysed with the HemosIL Factor VIII deficient plasma kit (Werfen), a one‐stage activity assay. Factor IX (FIX) activity was analysed with the HemosIL Factor IX deficient plasma kit (Werfen), a one‐stage activity assay. Von Willebrand Factor antigen (vWF ag) was analysed with the HemosIL von Willebrand Factor Antigen kit (Werfen), an automated latex enhanced immunoassay. Von Willebrand Factor activity (vWF a) was analysed with the HemosIL von Willebrand Factor Ristocetin Cofactor Activity kit (Werfen), an automated latex enhanced immunoassay. All tests were conducted according to the manufacturer's instructions.

### 2.4 *Data analysis*

Comparison of plasma pre and post pathogen‐inactivation treatment was performed using the two‐sample paired *t* test. Two‐tailed *p* values of <0.01 are considered statistically significant.

## 3 RESULTS

### 3.1 *The impact of plasma pooling on plasma volume standardisation*

Twenty‐five SDP units have been collected to generate five pools of five ABO‐identical SDP units respectively. Three pools were blood group B, one blood group A and one blood group O. The total volume loss during processing (pooling, pathogen inactivation, splitting) was 5.5 ± 1.9%. However, since six PTP units were produced from originally five SDP units, the total volume reduction per plasma unit was 21.1 ± 3.5%, an additional volume reduction of 15.6% (Table 1). The difference between the highest and lowest volume was reduced from 40 ml between SDPs to 22 ml between PTPs, a reduction of 46.5%.

**TABLE 1:** Volumes (ml) of the initially collected single donor plasma units (SDP), the total volume of the SDPs before pooling, the final pathogen‐reduced therapeutic plasma units (PTP), the total volume of the originally pooled units post processing (PP) and the total volume loss during processing (pooling, PI‐treatment, splitting) as well as the blood groups (BG) of each ABO‐identical pool

SDP number	Volume SDP (ml)	Pool number	Total volume (ml)	Volume PTP (ml)	Total volume PP (ml)	Volume loss PP (ml)	BG
1	258	1	1246	195	1170	76	O
2	259						
3	246						
4	234						
5	249						
6	250	2	1202	184	1104	98	A
7	232						
8	240						
9	232						
10	248						
11	268	3	1282	207	1242	40	B
12	248						
13	264						
14	263						
15	239						
16	250	4	1212	194	1164	52	B
17	232						
18	237						
19	243						
20	250						
21	261	5	1229	193	1158	71	B
22	251						
23	255						
24	233						
25	229						
Mean	247 ± 12		1234 ± 32	194 ± 8	1168 ± 49	69 ± 21	
Median	248		1229	194	1164	71	
Min	228		1202	184	1104	40	
Max	268		1282	207	1242	98	

*Note*: Mean values are expressed ± SD.

### 3.2 *The impact of plasma pooling on fibrinogen and labile coagulation factors standardisation*

The median fibrinogen content was 219 mg/dl (155–266) in SDPs and 177 mg/dl (168–179) in PTPs, a reduction of the spectrum (span between lowest and highest value) of 90.1%. The median FVIII content was 85 IU/dl (64–142) in SDPs and 70 IU/dl (49–87) in PTPs, a reduction of the spectrum of 51.3%. The median FIX content was 113 IU/dl (80–141) in SDPs and 86 IU/dl (70–96) in PTPs, a reduction of the spectrum of 57.4%. Median vWF activity was 75 IU/dl (47–120) in SDPs and 84 IU/dl (56–89) in PTPs, a reduction of the spectrum of 54.8% (Table 2). In total, the distribution of values found for the contents of fibrinogen and labile coagulation factors was reduced 63.4% in PTPs compared to SDPs.

**TABLE 2:** Variability of the content of labile coagulation factors and fibrinogen in single donor plasma units (SDPs) and pathogen‐reduced therapeutic plasma units (PTPs)

Factor	SDP unit		PTP unit	
	Content	Variability^a^	Content	Variability^a^
Fibrinogen (mg/dl)	219 (155–266)	111	177 (168–179)	11
Factor VIII (IU/dl)	85 (64–142)	78	70 (49–87)	38
Factor IX (IU/dl)	113 (80–141)	61	86 (70–96)	26
vWF a (IU/dl)	75 (47–120)	73	84 (56–89)	33

*Note*: The content columns are showing the median, minimum and maximum value.

Abbreviation: vWF a, von Willebrand factor activity.

^a^ Difference between unit with the lowest and the unit with the highest value.

### 3.3 *The impact of plasma pooling and pathogen‐inactivation treatment on the content of fibrinogen, labile coagulation factors and coagulation time*

The average fibrinogen retention was 78.7% with respect to the concentration (Table 3). However, the average total fibrinogen content per PTP unit was reduced 37.2 ± 2.5% compared to the SDP units, 24.6% due to processing and 12.6% due to volume reduction by splitting five units into six. The average FVIII retention was 67.8% with respect to the concentration. However, the average total FVIII content per PTP unit was reduced 42.1 ± 11.3% compared to the SDP units, 30.5% due to processing and 11.6% due to volume reduction by splitting five units into six. The average FIX retention was 74.8% with respect to the concentration. The average total FIX content per PTP unit was reduced 40.9 ± 2.6% compared to the SDP units, 29.1% due to processing and 11.8% due to volume reduction by splitting five units into six. Since the average vWF activity was not significantly different pre‐ and post‐treatment (*p* value 0.014) we also analysed the vWF antigen content, which was also not significantly different (*p* value 0.377). The average PT was prolonged for 5.2%, the average APTT for 14.5% (Table 3). The lowest FVIII, FIX and vWF concentrations were measured in the blood group O minipools as expected.

**TABLE 3:** Comparison of the fibrinogen and labile coagulation factor content as well as the coagulation time pre‐ and post PI‐treatment

Test	Pre‐PI	Post‐PI	Retention^a^	*p* value^b^
Fibrinogen (mg/dl)	221.4 ± 18.6	174.5 ± 4.8	78.7	<0.001
Factor VIII (IU/dl)	103.6 ± 16.1	70.2 ± 15.3	67.8	<0.001
Factor IX (IU/dl)	110.4 ± 13.4	82.6 ± 10.3	74.8	<0.001
vWF a (IU/dl)	83.6 ± 10.0	75.8 ± 15.0	90.7	0.014
vWF ag (IU/dl)	106.0 ± 13.4	90.0 ± 14.7	84.9	0.377
PT (s)	11.6 ± 0.2	12.2 ± 0.3	N/A	<0.001
APTT (s)	29.6 ± 2.1	33.9 ± 2.4	N/A	<0.001

*Note*: The values are showing the mean ± SD (*n* = 5).

Abbreviation: APTT, activated partial thromboplastin time; NA, not applicable; PT, prothrombin time; vWF a, von Willebrand factor activity; vWF ag, von Willebrand factor antigen.

^a^ Expressed in %.

^b^ Two‐tailed *p* value.

## 4 DISCUSSION

High inter‐donor variability in plasma factor content, coagulation time and variability in volumes of SDP doses could impact the clinical effectiveness of a plasma transfusion, hence the predictability of the treatment outcome. In the study presented here, the difference between the highest and lowest volume found was 46% less within the PTP units compared to the SDP units, the difference between highest and lowest concentration of fibrinogen and labile coagulation factors was reduced by 63%. A total reduction of variability of 55% (volume and coagulation factor concentration) between the units respectively was observed when the plasma units were produced by the novel approach of pooling five donor plasma units to gain six therapeutic plasma units in combination with AS pathogen inactivation. Moderate, but significant losses of coagulation factors (except for vWF) were observed post thawing of PTPs compared to fresh SDPs, findings which are in line with previously published data using amotosalen/UVA PI^12^, ^19^, ^20^ and in compliance with European standards.^21^ Also, a moderate prolongation of the coagulation time was observed, which is considered likely not being clinically relevant.

### 4.1 *Potential clinical benefits of reduced inter‐donor variability*

Better standardisation of therapeutic plasma units has major benefits in clinical practice. The treating physician ordering a unit for the treatment of a patient can rely much better on the therapeutic effect of the unit as he can expect that unit to contain comparable amounts of coagulation factors needed. A recent study reported significant inter‐donor variability in therapeutic plasma units in vitro, thus potentially impacting the protective effects of plasma‐based resuscitation during treatment of haemorrhagic shock in vivo, with potential implications for patient clinical outcome.^22^ But also, beyond the “standard” factors a standardised product has advantages. The authors of the above‐mentioned study furthermore concluded that also the concentration of certain chemokines in plasma has an impact on the mitigation of endothelial cell permeability. Higher levels of monocyte chemotactic protein 1 (MCP‐1), interleukin‐1 receptor agonist (IL‐1 Ra) and other chemokines were significantly increased in plasma units having a protective effect in haemorrhagic shock treatment (means reducing endothelial permeability). These findings show that inter‐donor variability may cause an uncertainty in treatment outcome which could potentially be mitigated by an increased standardisation of plasma units. Another example for inter‐donor variability impacting the clinical outcome is the presence of human leucocyte antigen (HLA) and human neutrophil antigen (HNA) antibodies in the plasma of single donors, causing transfusion‐related acute lung injury (TRALI), the leading cause of transfusion‐related mortality. Despite the introduction of protective measures, in particular deferral of high‐risk donors, there is still a remaining risk which could be mitigated (besides other measures) by plasma pooling, diluting the concentration of potentially harmful antibodies.^23^ However, a more predictable treatment outcome and increased safety by plasma pooling and PI does not necessarily translate into a better treatment outcome.

### 4.2 *Plasma pooling and safety concerns*

In many countries, SDP units are the standard of care. Historically the concerns regarding pooling have been the contamination of human plasma units with pathogens, for plasma particularly viruses, and to a certain degree, parasites. Current European guidelines allow the pooling of up to 12 human plasma units,^21^ reflecting the advancements in diagnostic screening in the last decades. Pathogen inactivation technology poses an additional layer of safety to reduce the risk of pathogen transmission. AS pathogen inactivation, which we evaluated in our study, efficiently inactivates blood borne viruses and other pathogens of concern in human plasma,^13^ also HIV, HBV and HCV (viruses which could be transmitted despite the usage of state of the art screening tests during a window period).

### 4.3 *Economical and operational considerations*

The pooling of five SDP units resulting in six PTPs concept allows for the production of a more standardised product with potentially increased predictability of the clinical treatment outcome (which needs to be evaluated further in clinical studies), with a potential to reduce overall treatment costs by reducing inefficient treatment and adverse reactions. Due to the pathogen inactivation treatment of pools, only two pathogen inactivation processing sets are used to manufacture six PTPs. The additional cost of those two sets and the pooling set could be partially mitigated by the generation of six PTP units from five SDP units, allowing additional economic benefits. The current COVID‐19 pandemic demonstrated how quickly a new pathogen could spread, and how quickly borders close and the exchange of products could become a challenge. Independence of the blood transfusion centres in generating pathogen‐reduced pooled plasma from their donors locally poses an important feature of preparedness to guarantee an independent blood supply in case of future pandemics or other adverse reactions. In the Polish guidelines updated in 2020 the procedure of plasma pooling and inactivation is approved.

### 4.4 *Conclusion*

The pooling of five SDP units to obtain six PTP units significantly increases plasma standardisation with potential implications for safety of the transfusion recipient, making it an interesting alternative to qSDP and pathogen‐reduced SDP, especially in the light of pandemic preparedness.

## CONFLICT OF INTEREST

Marcus Picard‐Maureau is employee of Cerus Europe B.V., the manufacturer of the INTERCEPT Blood System. All other authors declare no conflict of interest.

## AUTHOR CONTRIBUTIONS

**Michal Bubinski**, **Pawel Szykula** and **Kamila Kluska:** Performed the research study. **Michal Bubinski**, **Agnieszka Gronowska**, **Marcus Picard‐Maureau** and **Elzbieta Lachert:** Designed the research study. **Ilona Kuleta** and **Elzbieta Lachert:** Contributed to essential reagents and tools. **Michal Bubinski**, **Agnieszka Gronowska** and **Marcus Picard‐Maureau:** Analysed the data. **Michal Bubinski**, **Elzbieta Lachert** and **Marcus Picard‐Maureau:** Wrote the manuscript. All authors reviewed and approved the final draft.
